# Task-Aligned Speech Representations for Intelligibility Classification in Patients with Cleft Palate

**DOI:** 10.3390/s26216975

**Published:** 2026-11-04

**Authors:** Xiao Luo, Shan Liao, Xue Gong, Zhenyu Liu, Xueni Zhao, Chenghao Li, Ling He, Heng Yin

**Affiliations:** 1 State Key Laboratory of Oral Diseases & National Clinical Research Center for Oral Diseases & Department of Cleft Lip and Palate Surgery, West China School of Stomatology, Sichuan University, Chengdu 610041, China; luox@stu.scu.edu.cn (X.L.); leechenghao@scu.edu.cn (C.L.); 2 College of Biomedical Engineering, Sichuan University, Chengdu 610065, China; hd6445@outlook.com (S.L.); gong_xue@stu.scu.edu.cn (X.G.); 2025141670070@stu.scu.edu.cn (Z.L.); ling.he@scu.edu.cn (L.H.); 3 Department of Otorhinolaryngology, Head and Neck Surgery, The Chinese University of Hong Kong, Shatin, New Territories, Hong Kong SAR 999077, China; 1155278960@link.cuhk.edu.hk

**Keywords:** cleft palate, speech intelligibility, HuBERT, self-supervised learning, pathological speech, acoustic sensing

## Abstract

Perceptual evaluation remains the clinical standard for postoperative cleft-palate speech, but objective patient-level assessment of overall speech intelligibility remains limited. We developed a patient-level three-class classification model using frozen Chinese HuBERT representations, principal component analysis (PCA), and regularized multinomial logistic regression. The study included 123 patients with cleft palate, including 100 in the development cohort and 23 in the held-out cohort. Each patient completed 27 standardized articulation tasks. HuBERT embeddings from each task were summarized by temporal mean and standard deviation and concatenated in a fixed clinical order to form a patient-level representation. Model development used patient-disjoint nested cross-validation followed by locked held-out evaluation. The primary model achieved 90.00% accuracy and a macro-F1 of 0.8659 in development and 86.96% accuracy, a macro-F1 of 0.8227, 93.75% balanced accuracy, and a quadratic-weighted kappa of 0.9043 in the held-out cohort. All three held-out errors involved patients with Poor intelligibility who were misclassified as Reduced intelligibility, with no direct Normal-to-Poor errors. Comparisons across six classifiers and four pretrained encoders showed no significant paired differences after Holm correction. Task-replacement analysis revealed heterogeneous reliance on individual articulation tasks. These findings support the feasibility of objective patient-level speech intelligibility assessment from standardized cleft-palate speech recordings.

## 1. Introduction

Cleft palate (CP) is a congenital craniofacial anomaly that may result in persistent speech impairment even after primary palatal repair. Postoperative speech abnormalities commonly include resonance disorders, nasal emission, and compensatory articulation, which often coexist and collectively reduce overall speech intelligibility [1,2]. Reliable assessment of postoperative speech is therefore important for evaluating treatment outcomes and guiding further speech therapy or secondary surgical intervention. Perceptual evaluation by experienced speech-language pathologists remains the clinical gold standard for cleft palate speech assessment [3,4,5]. However, perceptual ratings are influenced by listener experience and speech-sample characteristics. Moreover, access to experienced cleft speech specialists is often limited, which can restrict timely and standardized assessment during postoperative follow-up. These limitations have encouraged the development of objective computational methods for assessing speech intelligibility in patients with cleft palate [6].

Automatic assessment of speech intelligibility has progressed from handcrafted acoustic descriptors and conventional machine-learning classifiers to deep neural models, automatic speech recognition (ASR)-derived metrics, and pretrained or self-supervised speech encoders [7,8,9,10,11,12,13,14,15,16,17]. However, advances in speech representation learning have not yet been fully translated into clinically integrated assessments of cleft palate speech. Most previous studies have instead focused on individual abnormalities, such as hypernasality, articulation errors, or velopharyngeal dysfunction, rather than overall speech intelligibility [18,19,20,21,22,23,24,25,26,27,28]. In addition, standardized cleft palate speech assessments comprise multiple predefined phonetic tasks, yet how task-specific speech information can be integrated into patient-level representations for overall speech intelligibility assessment remains insufficiently explored. This issue may be particularly relevant to Mandarin, in which language-specific syllabic and phonetic structures influence the acoustic manifestation of resonance and articulation abnormalities [29].

Accordingly, this study focuses on patient-level classification of overall speech intelligibility in postoperative Mandarin-speaking patients with cleft palate. The evaluation target is the patient-level intelligibility category derived from a standardized Mandarin articulation assessment comprising 27 predefined phonetic tasks, rather than an isolated speech abnormality or a single recording. Task-level representations extracted using a frozen Chinese HuBERT encoder were organized according to the predefined clinical task structure, preserving the correspondence between each acoustic representation and its elicitation condition before patient-level integration. Unlike conventional approaches that directly aggregate speech representations without considering task identity, our framework constructs a task-aligned patient-level representation that captures speech characteristics across standardized phonetic contexts. Model development used patient-disjoint nested cross-validation, followed by evaluation on a locked held-out cohort. The methodological contribution of this study therefore does not involve developing a new speech encoder or classifier, but rather establishing a clinically structured representation framework that leverages pretrained speech representations for patient-level intelligibility assessment.

To examine the modeling choices within this framework, six classifier families and four pretrained speech encoders were evaluated under predefined comparison protocols. Patient-level prediction probabilities and speech-task replacement analyses were further used to characterize error patterns and model reliance. The main contributions of this study are:

1. We formulate postoperative cleft-palate speech assessment as a patient-level three-class intelligibility classification problem, targeting overall speech intelligibility rather than an isolated speech abnormality or a single utterance.
2. We introduce a task-aligned patient representation framework that integrates acoustic information from 27 standardized articulation tasks while preserving the correspondence between each task-level representation and its clinical elicitation condition. This design enables patient-level modeling of speech patterns across multiple phonetic contexts rather than treating task-derived representations as an unordered feature collection.
3. We establish a leakage-controlled patient-level evaluation framework using patient-disjoint nested cross-validation and a locked held-out cohort, complemented by task-replacement analysis to characterize task dependence and patient-specific prediction patterns.

## 2. Related Work

### 2.1. Acoustic Features and Conventional Machine Learning

Early automatic speech intelligibility assessment relied primarily on handcrafted acoustic descriptors designed to characterize deviations from typical speech production. Common features included Mel-frequency cepstral coefficients (MFCCs), spectral and cepstral measures, prosodic variables, glottal parameters, and articulatory or phonatory descriptors. These features were combined with conventional classifiers or regression models, including support vector machines, probabilistic linear discriminant analysis, artificial neural networks, and k-nearest-neighbor methods. Both reference-free and reference-based strategies have been investigated: reference-free approaches estimate intelligibility directly from impaired speech, whereas reference-based approaches quantify deviations from a reference signal before mapping them to an intelligibility level [8].

Because acoustic descriptors are often extracted at the frame level, aggregation strategies have also been used to construct fixed-dimensional representations for intelligibility assessment. For example, Fisher-vector encoding of frame-level spectral and cepstral features improved utterance-level dysarthric intelligibility classification relative to simpler temporal encoding schemes [9]. Collectively, these studies established the feasibility of automated intelligibility grading while highlighting the influence of feature engineering, speaker variability, and evaluation design. Previous work has also reported lower performance under speaker-independent evaluation than under random sample-level splitting [8], underscoring the importance of evaluating generalization to unseen individuals.

### 2.2. Neural Network-Based Intelligibility Modeling

Neural network-based methods shifted the emphasis from manually selected acoustic descriptors toward representations learned directly from speech data. Convolutional neural networks (CNNs) capture local time-frequency patterns from spectrograms, whereas recurrent and long short-term memory (LSTM) architectures model temporal dependencies across speech frames. Spectro-temporal CNNs have shown that jointly modeling frequency and temporal variation can improve dysarthric intelligibility assessment compared with representations emphasizing only one dimension [7]. Latent-representation approaches have further attempted to separate intelligibility-related information from speaker identity and linguistic content [10]. Multi-objective neural models have also broadened the assessment target: MOSA-Net jointly estimates speech quality, intelligibility, and distortion-related measures, whereas TorchAudio-SQUIM provides reference-free estimates of established speech-quality and intelligibility metrics [12,13]. These studies established the value of task-specific neural modeling for pathological speech assessment and motivated the use of learned representations beyond handcrafted acoustic features.

### 2.3. ASR and Pretrained Speech Representation Approaches

Automatic speech recognition (ASR)-based assessment provides a complementary strategy in which recognition errors serve as proxies for how understandable a speech sample is to a listener. Word recognition rate, word error rate, and phoneme-level errors can therefore be used as automatically generated intelligibility indices. Clinical studies have shown that generic ASR output is related to, but not interchangeable with, human intelligibility judgments [11], and clinically adapted ASR-derived measures have subsequently been evaluated across neurological disorders and languages [15]. In parallel, self-supervised and other pretrained speech encoders learn general acoustic and phonetic representations from large external speech corpora before application to smaller clinical datasets. Wav2Vec2-based representations have been used to predict intelligibility and severity from limited pathological-speech samples [14], while pretrained wav2vec 2.0 and HuBERT embeddings have outperformed conventional acoustic features in dysarthria detection and severity classification [16]. A cross-language Wav2Vec2 approach has also derived articulatory-precision measures associated with perceptual intelligibility across multiple languages [17]. Unlike the task-specific neural models summarized above, these approaches exploit representations learned from large-scale external speech data; their performance may depend on encoder choice, representation extraction, language match, and evaluation design.

### 2.4. Cleft Palate Speech Intelligibility Assessment

Compared with dysarthric and other pathological speech, relatively few studies have directly modeled overall speech intelligibility in cleft palate. Early work showed that automatic speech recognition-derived measures were associated with expert intelligibility judgments in children with cleft lip and palate [6]. Subsequent research has focused predominantly on specific speech abnormalities, particularly hypernasality, articulation errors, and velopharyngeal-dysfunction-related speech characteristics [18,19,20,21,22,23,24,25,26,27,28,29,30]. A recent systematic review similarly found that AI-based cleft-speech research remains dominated by component-level targets rather than integrated speech outcomes [30]. Consequently, patient-level assessment of overall cleft-palate speech intelligibility remains comparatively underexplored, especially when speech information is obtained from standardized multi-task articulation protocols.

## 3. Materials and Methods

### 3.1. Participants and Dataset

The Huaxi Cleft Palate Speech Intelligibility (HCPSI) dataset was collected at the West China School of Stomatology, Sichuan University, during standardized postoperative speech assessment. The initial HCPSI dataset comprised 130 patients, including 104 assigned to the development cohort and 26 to the held-out test cohort.

Patients were eligible if they had undergone cleft palate repair and completed the standardized Mandarin speech assessment with an expert-consensus intelligibility label. Among eligible patients, cases were excluded from the present analysis if one or more of the 27 predefined core articulation tasks were missing, substantial recording noise prevented reliable evaluation, or speech intelligibility could not be reliably graded by the clinical raters. When repeated recordings or multiple assessment records were available for the same patient, only one patient-level record was retained to maintain statistical independence.

Following these quality-control procedures, four development patients were excluded because their recordings were ungradable or contained substantial noise, and three held-out patients were excluded because required speech tasks were missing. These patients were not replaced after quality control, thereby preserving the predefined locked status of the held-out cohort. The final evaluable dataset therefore comprised 123 patients: 100 in the development cohort and 23 in the locked held-out cohort. The distributions of the three reference categories (Normal intelligibility, Reduced intelligibility, and Poor intelligibility) are presented separately in Table 1.

The final cohort had a median age of 14 years (range, 4–37 years) and included 63 male and 60 female patients. The median age was 14 years (range, 4–37 years) in the development cohort and 12 years (range, 4–35 years) in the held-out cohort. The development cohort included 48 male and 52 female patients, whereas the held-out cohort included 15 male and 8 female patients. No significant between-cohort differences were observed in age (*p* = 0.582) or sex distribution (*p* = 0.136). Demographic characteristics are summarized in Table 2.

### 3.2. Speech Recording and Standardized Articulation Protocol

Speech recordings were obtained during standardized clinical assessment in a professional soundproof recording room at the Speech Therapy Center of West China Hospital of Stomatology, Sichuan University. All recordings included in the present analysis were acquired using a LEWITT LCT940 microphone (LEWITT LCT940 microphone, Vienna, Austria) and an RME UCX audio interface (IMM Elektronik GmbH, Mittweida, Germany) at an original sampling rate of 44.1 kHz. For subsequent representation extraction, recordings were converted to mono and resampled to 16 kHz when required by the pretrained speech encoder.

Each patient completed 27 standardized articulation tasks that constitute a predefined core component of the Mandarin speech assessment protocol used at our center. These tasks were not selected retrospectively according to model performance. The task set was designed to cover the 21 Mandarin initials and six basic finals while sampling phonetic contexts relevant to common cleft-palate speech errors across different places and manners of articulation. Each word was treated as a fixed articulation task representing a predefined phonetic condition.

### 3.3. Clinical Reference Standard

The reference standard for patient-level speech intelligibility was established by three clinicians experienced in cleft-palate speech assessment, each with more than 10 years of clinical experience. All three clinicians independently evaluated the standardized speech samples of each patient and assigned the patient to one of three clinically defined categories: Normal intelligibility, Reduced intelligibility, or Poor intelligibility. The three ratings were subsequently compared. Cases with discrepant initial ratings were jointly reviewed and discussed until consensus was reached, and the consensus category, rather than any score-derived label, was used as the final reference label for all subsequent analyses. Inter-rater agreement among the three clinicians’ independent ratings was assessed using Fleiss’ kappa and was substantial (κ = 0.719).

All perceptual assessments were completed before development of the present computational model. The assessors therefore had no access to model predictions and were unaware of the subsequent assignment of patients to the development or held-out cohort.

### 3.4. Overview of the Patient-Level Modeling Framework

The modeling framework retained the standardized 27-task structure throughout patient-level feature construction. Each recording remained associated with its predefined articulation task, ensuring that the same task position referred to the same clinical elicitation condition across patients.

**Figure 1:**
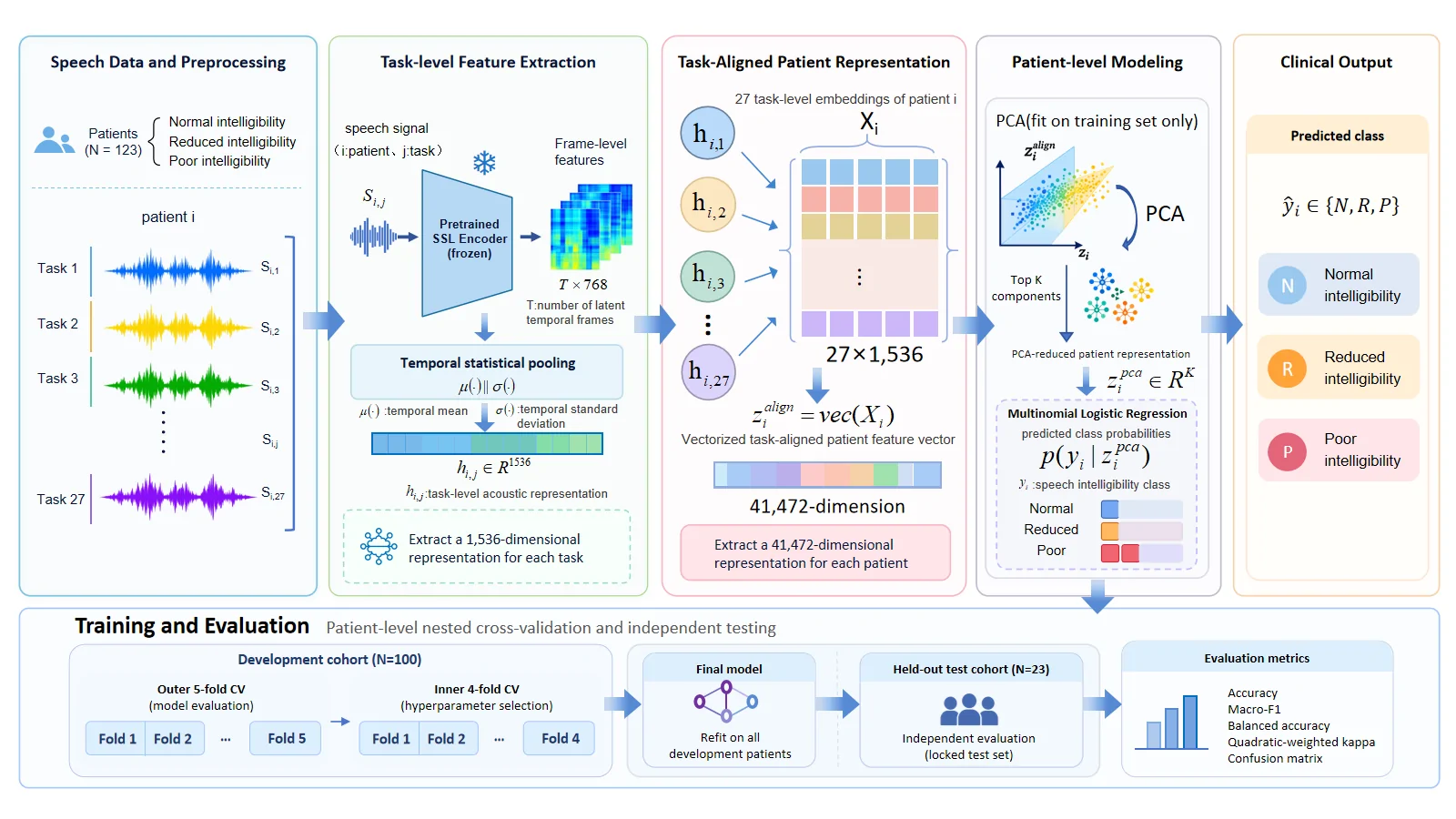
Overview of the task-aligned patient-level speech intelligibility classification framework.

As illustrated in Figure 1, each recording was independently encoded using a frozen pretrained speech encoder and temporal statistical pooling to obtain a fixed-dimensional task-level vector. The 27 task-level vectors were then arranged in the predefined clinical order, vectorized into a 41,472-dimensional patient-level representation, and passed to training-set standardization, principal component analysis (PCA), and multinomial logistic regression for three-class intelligibility prediction.

All data-dependent preprocessing, dimensionality reduction, model selection, and classifier fitting were performed within the corresponding patient-level training partitions. Model performance was evaluated using patient-disjoint nested cross-validation in the development cohort, followed by independent evaluation on a locked held-out test cohort.

### 3.5. Task-Level Acoustic Representation

To obtain a robust acoustic representation from the limited clinical dataset, a pretrained self-supervised speech encoder was employed exclusively as a fixed feature extractor. The encoder was not fine-tuned on the CP dataset. This design separates the extraction of general-purpose speech representations from the subsequent modeling of the clinical phenotype and avoids introducing a large number of trainable parameters into the small clinical cohort.

For the j-th speech task of patient i, let the input waveform be denoted by s_(i,j)(t). The waveform is first transformed into a sequence of latent acoustic features through a convolutional feature extraction operator:

(1) Z^(0)^_i_,_j_ = f_conv(s_i_,_j_)

where Z^(0)^_i_,_j_ ∈ R^(T_i_,j × d_0_), and T_i_,_j_ denotes the number of latent temporal frames. The resulting sequence is subsequently processed by a stack of contextual encoding layers:

(2) Z^(l)^_i_,_j_ = T_θ_l_(Z^(l−1)^_i_,_j_), l = 1, …, L

where T_θ_l_ denotes the l-th contextual encoding operator and L is the total number of encoding layers. The final contextual representation is given by:

(3) H_i_,_j_ = Z^(L)^_i_,_j_ = f_θ(s_i_,_j_)

with H_i_,_j_ ∈ R^(T_i_,_j_ × 768). The encoder parameters θ were fixed throughout the study. Consequently, all patient-specific learning occurred downstream of the pretrained representation space.

Because the duration of individual speech recordings varies across tasks and patients, a temporal aggregation operator was used to convert the frame-level representation into a fixed-dimensional task-level vector. For the k-th feature dimension, the temporal mean was defined as:

(4) μ_i_,_j_^k^ = (1/T_i_,_j_) Σ_t=1_^Ti,j^ H_i_,_j_^(t,k)

while the corresponding temporal standard deviation was calculated as:

(5) σ_i_,_j_^k^ = √[(1/T_i_,_j_) Σ_t=1_^Ti,j^ (H_i_,_j_^(t,k) − μ_i_,_j_^k^)^2^]

The task-level acoustic representation was obtained by concatenating the two statistics:

(6) h_i_,_j_ = [μ_i_,_j_; σ_i_,_j_] ∈ R^1536^

Thus, each standardized speech task was represented by a 1536-dimensional vector summarizing both contextual acoustic characteristics and temporal variability. This operation was performed independently for each task before patient-level aggregation, thereby maintaining correspondence between each acoustic representation and the speech task from which it was derived.

### 3.6. Task-Aligned Patient Representation

The central methodological component was the transformation of task-level speech embeddings into a structured patient-level representation. Rather than treating the 27 task embeddings as an unordered collection, their predefined clinical correspondence was preserved.

For patient i, the 27 task-level embeddings were arranged according to the standardized assessment order:

(7) X_i_ = [h_i_,_1_^T^; h_i_,_2_^T^; …; h_i_,_27_^T^] ∈ R^27×1536^

The j-th row of X_i therefore corresponded to the same clinical elicitation task for every patient. This representation established a structured mapping between task-specific acoustic characteristics and patient-specific speech characteristics. In other words, each patient was represented by a profile describing how acoustic characteristics were expressed across the same set of standardized speech conditions.

The matrix representation was subsequently vectorized to obtain the patient-level task-aligned feature vector:

(8) z^align^_i_ = vec(X_i_) ∈ R^(27 × 1536) = R^41472^

The dimensionality of z^align^_i_ is therefore determined by the number of standardized clinical tasks and the dimensionality of the task-level acoustic representation. This representation retains each task-level feature vector in a separate, predefined block, whereas mean pooling averages across tasks. This structural distinction does not, by itself, establish an independent performance benefit of preserving task identity.

Accordingly, the vectorized task-aligned representation was used as the patient-level input for all subsequent modeling steps:

(9) z_i_ = z^align^_i_ ∈ R^41472^

Conceptually, this representation describes each patient as a structured profile of task-dependent speech behavior across the same standardized elicitation conditions, providing a consistent coordinate system for comparing individual speech patterns.

### 3.7. Leakage-Free Patient-Level Model Development

The resulting task-aligned representation contains 41,472 dimensions, substantially exceeding the number of available patients. Such a high-dimensional, low-sample-size setting introduces a substantial risk of overfitting and information leakage if preprocessing and model selection are performed using the complete cohort. We therefore adopted a nested patient-level cross-validation framework in which every data-dependent operation was restricted to the corresponding training partition.

The development dataset was divided into five outer folds at the patient level. During each outer iteration m, four folds were used as the outer training set, D_train^(m)^, and the remaining fold was retained as the outer validation set, D_val^(m)^. The outer validation set was not used for feature transformation, dimensionality reduction, hyperparameter selection, or classifier fitting.

Within each outer training set, four-fold inner cross-validation was used to select the model configuration. The hyperparameter search included the dimensionality of the PCA representation, the regularization strength of the classifier, and the class weighting strategy. For a candidate hyperparameter configuration λ, the optimal configuration was determined exclusively from the inner-fold performance:

(10) λ* = arg max_λ S_inner(λ)

where S_inner denotes the selected performance criterion calculated from the inner validation folds.

All feature transformations were fitted independently within the corresponding training data. Let μ^(m)^ and σ^(m)^ denote the feature-wise mean and standard deviation estimated from the outer training set. Standardization was performed as:

(11) $${\tilde{z}}_{i}=(z_{i}-\mu^{\left(m\right)})/\sigma^{\left(m\right)}$$

The standardized representations were subsequently projected into a lower-dimensional space using PCA fitted only on the outer training data. If U_d^(m)^ denotes the matrix containing the first d principal directions, the reduced representation is:

(12) $${r_{i}}^{\left(m\right)}={U\_d}^{(m)T}\;{\tilde{z}}_{i}$$

Neither the standardization parameters nor the PCA directions were estimated using the outer validation patients.

Following inner-fold model selection, the complete outer training set was used to refit the preprocessing and classification pipeline using the selected configuration. The resulting model can be expressed as:

(13) M^(m)^ = g_φ* ∘ PCA_d*^(m)^ ∘ Scaler^(m)^

where g_φ* denotes the optimized classifier and d* denotes the selected PCA dimensionality. Predictions for patients in the outer validation fold were then generated as:

(14) ŷ_i_^(m)^ = M^(m)^(z_i_)

Predictions from all five outer folds were subsequently aggregated to obtain out-of-fold predictions for the complete development cohort. These predictions were generated exclusively from models that had not encountered the corresponding patients during training or hyperparameter optimization.

After model development was completed, the selected modeling configuration was refitted using the entire development data. The locked test dataset was then processed using the finalized pipeline and evaluated once. Thus, the test dataset did not contribute to any development decision, including feature preprocessing, PCA dimensionality selection, regularization selection, or class weighting.

This nested design establishes a strict separation between model development and performance estimation and is particularly important for the proposed high-dimensional representation, where seemingly minor preprocessing leakage can otherwise result in optimistic performance estimates.

### 3.8. Classification and Statistical Analysis

Regularized multinomial logistic regression was adopted as the primary downstream classifier because it provides a low-complexity and interpretable modeling framework appropriate for the limited clinical sample size. Given a reduced patient representation r_i_, the probability of class c was modeled using the softmax function:

(15) p(y_i_ = c | r_i_) = exp(w_c^T^r_i_ + b_c)/Σ_k=1_^3^ exp(w_k^T^r_i_ + b_k), c ∈ {1, 2, 3}

where w_c and b_c denote the coefficient vector and intercept for class c, respectively. Regularization was incorporated to constrain model complexity in the high-dimensional representation space.

The task-aligned patient representation was used for all primary analyses, with a task-agnostic mean-pooling baseline included for comparison.

Model performance was evaluated using accuracy, balanced accuracy, macro-averaged F1-score, and quadratic-weighted kappa (QWK). Development-cohort performance was estimated from the aggregated outer-fold predictions, while the locked held-out cohort was used for final evaluation.

Where reported, uncertainty intervals were calculated at the patient level. Wilson 95% intervals were used for held-out accuracy, and bootstrap procedures were used for exploratory patient-level comparisons and macro-F1 uncertainty as specified below.

### 3.9. Experimental Setup

Each patient was represented by 27 aligned speech tasks. The main pipeline used a frozen Chinese HuBERT Base encoder (TencentGameMate/chinese-hubert-base). Waveforms were converted to mono, centered, and resampled to 16 kHz when necessary. Within each task, the temporal mean and population standard deviation of the final 768-dimensional hidden representation were concatenated, giving 1536 features. Concatenation in a fixed task order yielded 41,472 patient-level features. The main experiment used the archived batched extraction pipeline with batches of 16, padding, attention masks, and masked temporal pooling. File names were used only for task alignment and were not encoded as predictive features. The encoder was not fine-tuned.

Performance on the development dataset was assessed using a fixed, patient-disjoint nested cross-validation scheme. The five outer folds contained 20, 19, 20, 20, and 21 patients. Within each outer training partition, four-fold inner cross-validation was used for hyperparameter selection. The exact patient counts for each outer and inner split are presented in Table 3.

Feature standardization and randomized principal component analysis (PCA) were fitted exclusively to the corresponding training subset. Within each training partition, randomized PCA was fitted with 20 components, from which either the first 10 or all 20 components were retained according to the candidate configuration. Candidate PCA dimensionalities were 10 and 20. Multinomial logistic regression (LR) was evaluated with inverse regularization strengths C in {0.001, 0.01, 0.1, 1} and either uniform or balanced class weights, giving 16 configurations. An exhaustive grid search was implemented by enumerating all 16 predefined combinations of PCA dimensionality, regularization strength, and class weighting. Each configuration was evaluated using the same patient-disjoint inner-validation folds, with standardization and PCA fitted exclusively on the corresponding training data. Hyperparameters were selected based on macro-F1 calculated from pooled inner-validation predictions, with accuracy, quadratic-weighted kappa (QWK), fewer retained PCA components, smaller C values, and a predefined configuration order used as successive tie-breaking criteria. The random seed for PCA and the classifier was set to 20260906.

For held-out evaluation, a separate five-fold search using only the 100 development patients selected the final configuration, which was then refitted on all development patients. The final main model used 10 principal components, C = 0.01, and balanced class weights. Its predictions were locked before evaluation. The configuration-selection cross-validation score was not treated as an additional unbiased validation estimate; the reported development results below are the nested outer-fold predictions. All recordings from a patient remained in a single partition.

Feature extraction used an NVIDIA GeForce RTX 4060 Laptop GPU with 8 GB memory, PyTorch 2.6.0 + cu124, and Transformers 5.16.1. Classifiers were implemented with scikit-learn 1.9.0. GPU inference used automatic FP16 mixed precision with FP32 temporal pooling.

### 3.10. Evaluation Measures and Comparisons

The principal measures were patient-level accuracy and macro-F1. Balanced accuracy, QWK, class-wise recall, and confusion matrices were also reported. Macro-F1 assigns equal weight to the three classes, whereas balanced accuracy averages class-wise recall. The always-majority-class accuracy was 62.00% on development and 69.57% on testing. Wilson 95% intervals were calculated for test accuracy. Exploratory paired bootstrap intervals used 10,000 resamples of fixed patient-level out-of-fold predictions; these intervals do not incorporate retraining variability and were not adjusted for multiple comparisons. No universal superiority claim was based solely on overlapping point estimates.

Two comparisons were conducted. First, six classifier families were evaluated using the same archived HuBERT features: LR, linear SVM, RBF SVM, random forest, k-nearest neighbors (KNN), and a single-hidden-layer multilayer perceptron (MLP). Each classifier family was independently optimized over 16 predefined configurations using the patient-disjoint nested cross-validation and grid-search procedure described above. For logistic regression and linear SVM, the inverse regularization strength C was searched over {0.001, 0.01, 0.1, 1}, with class weights selected from {None, balanced}. For RBF SVM, C was searched over {0.1, 1, 10, 100} with the same class-weight options and gamma fixed as scale. Random forest configurations varied max_depth ∈ {3, None}, min_samples_leaf ∈ {1, 3}, and class weights ∈ {None, balanced}, with 300 trees and max_features set to sqrt. KNN configurations included n_neighbors ∈ {3, 5, 9, 15} and uniform or distance weighting. The MLP used a single hidden layer with either 16 or 32 units and alpha ∈ {0.001, 0.01, 0.1, 1}, with ReLU activation and L-BFGS optimization. The MLP additionally standardized PCA scores within each training subset. Equal candidate counts did not constitute exhaustive optimization of each family.

Second, the feature extractor was replaced by Chinese wav2vec 2.0 Base, WavLM Base+, or data2vec Audio Base while retaining the same PCA/LR search. Each encoder was evaluated independently using the same 16-configuration grid-search and patient-level nested cross-validation procedure described in Section 3.9. For held-out evaluation, each final model was selected using only the development cohort and refitted on all 100 development patients. For this comparison, all four encoders, including HuBERT, were evaluated with newly extracted single-utterance features, without padding or truncation. The same final-layer mean/standard-deviation pooling and task order were used. This separate extraction protocol avoids mixing different batch-padding treatments across encoders, but differs from the main archived pipeline. This encoder comparison must therefore be interpreted against its newly extracted HuBERT baseline, not against the main 90% development result. The checkpoints also differ in pretraining language and corpus size; the comparison measures available pretrained representations, not a controlled causal effect of architecture alone.

Additional retrospective uncertainty analyses were performed on the fixed test predictions. Macro-F1 intervals used 10,000 class-stratified patient bootstrap resamples (seed 20260917), retaining the observed class counts of 5, 2, and 16. These percentile intervals are conditional on this class composition and do not capture model-training or selection uncertainty. Paired correctness was compared using two-sided exact McNemar tests against the within-round reference (LR for the classifier comparison; newly extracted HuBERT for the encoder comparison). Holm adjustment was applied jointly across all eight comparisons. These post hoc tests were exploratory; correction for these eight comparisons does not undo the broader history of dataset reuse.

### 3.11. Post Hoc Patient-Level and Speech-Task Analyses

Post hoc analyses were conducted using the locked primary HuBERT/PCA/logistic-regression model and all 23 patients in the held-out cohort. No model parameters, decision rules, patient eligibility criteria, or reference labels were modified during these analyses. Patient-level prediction patterns were examined using the three class probabilities generated by the fitted model. These probabilities were treated as raw classifier outputs and were not interpreted as calibrated clinical confidence estimates.

To characterize the model’s reliance on individual standardized speech tasks, a task-replacement analysis was performed. For each held-out patient, each 1536-dimensional task block was replaced, one task at a time, by the corresponding mean feature block calculated from the 100-patient development cohort. The remaining 26 task blocks and the fitted standardizer, principal component analysis (PCA) transformation, and logistic-regression classifier were left unchanged.

For patient i and task t, the signed replacement effect was defined as Δ_i_,_t_ = p_i_,_yi_(original) − p_i_,_yi_(−t), where y_i_ denotes the reference intelligibility class, p_i_,_yi_(original) is the predicted probability assigned to the reference class using the original patient representation, and p_i_,_yi_(−t) is the corresponding probability after replacement of task t. A positive value therefore indicates that replacing the task reduced the reference-class probability, whereas a negative value indicates that replacement increased the reference-class probability.

To account for class imbalance in the held-out cohort, signed replacement effects were first averaged separately within each reference class. The unweighted mean of the three class-specific effects was then calculated as the class-balanced task effect and used for descriptive task ranking.

For case-level visualization, all misclassified held-out patients were retained. One correctly classified comparison case from each reference class was selected deterministically as the patient with the lowest pseudonymized identifier within that class. This selection procedure was independent of the observed task-replacement effects. The resulting patient-level and task-level analyses were intended to characterize prediction patterns and model reliance under the specified replacement baseline rather than to provide causal estimates of individual task importance.

## 4. Results

### 4.1. Task-Aligned Versus Task-Agnostic Representation

To evaluate the contribution of task-aligned representation, we compared the proposed framework with a task-agnostic mean-pooling baseline under identical patient-level evaluation protocols. As shown in Table 4, the task-aligned approach achieved higher performance across all reported metrics in both the development and held-out cohorts.

In the development cohort, task-aligned representation achieved 90.00% accuracy and a macro-F1 of 0.8659, compared with 82.00% and 0.7513 for mean pooling, respectively. A similar trend was observed in the held-out cohort, where task-aligned representation achieved a macro-F1 of 0.8227, compared with 0.7012 for mean pooling.

These results show higher performance point estimates for task-aligned concatenation than for mean pooling under the evaluated protocol. They reflect a comparison of the two patient-representation strategies as a whole, rather than an isolated evaluation of task identity.

### 4.2. Automatic Assessment of Speech Intelligibility

Direct three-class training correctly classified 90 of 100 development patients, corresponding to 90.00% accuracy, a macro-F1 of 0.8659, balanced accuracy of 85.51%, and QWK of 0.8519. On the 23 held-out patients, the main model correctly classified 20 patients, yielding 86.96% accuracy (Wilson 95% CI, 67.87–95.46%), a macro-F1 of 0.8227 (95% CI, 0.7231–1.0000), balanced accuracy of 93.75% (95% CI, 87.50–100.00%), and QWK of 0.9043 (95% CI, 0.8078–1.0000) (Table 5).

**Figure 2:**
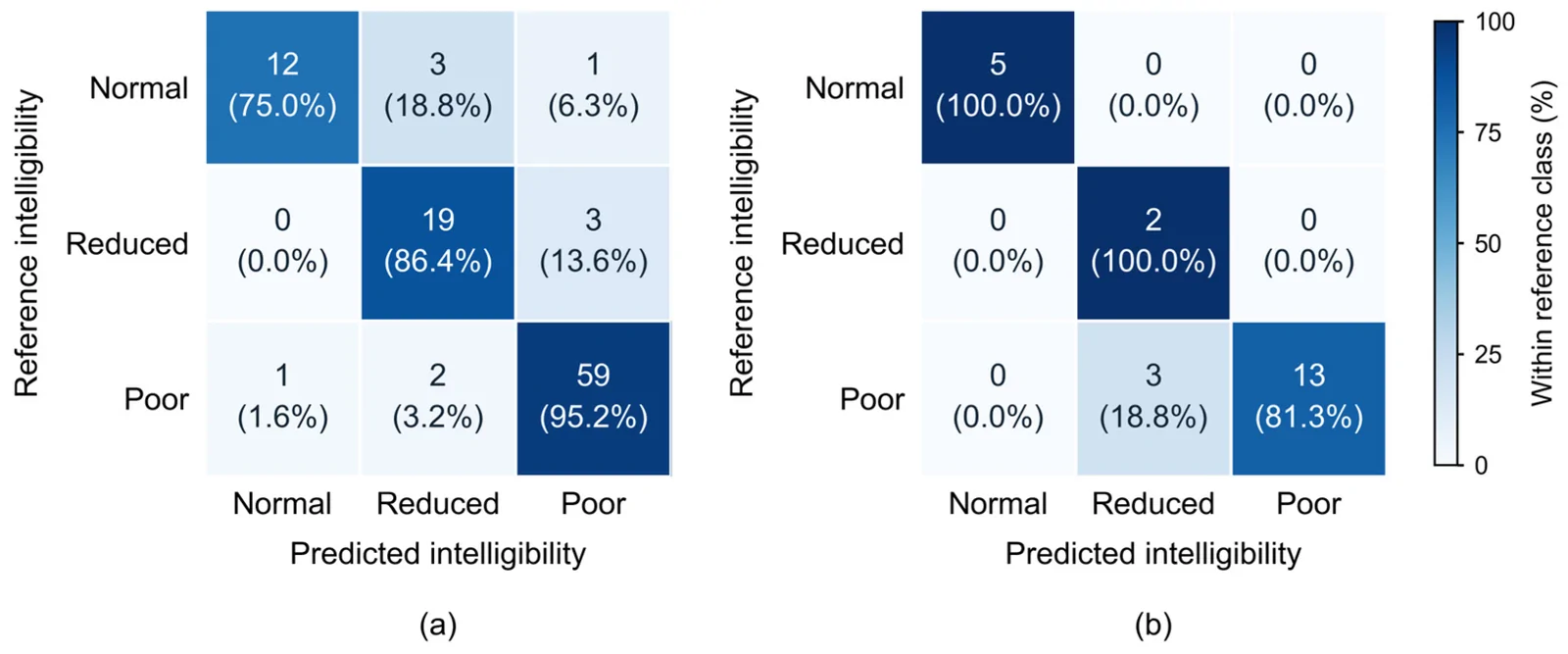
Patient-level confusion matrices for (**a**) the development evaluation and (**b**) the held-out evaluation.

All five held-out patients with Normal intelligibility and both patients with Reduced intelligibility were correctly classified. Thirteen of 16 patients with Poor intelligibility were correctly classified; the remaining three were predicted as Reduced intelligibility. From a clinical grading perspective, all held-out errors were confined to the boundary between Reduced and Poor intelligibility, with no direct confusion between Normal and Poor intelligibility. Although recall for Reduced intelligibility was 100%, precision was 40.00% because the three errors from the Poor category increased the number of Reduced predictions. However, the 100% recall for Reduced intelligibility was based on only two patients, with a wide exact 95% confidence interval (15.81–100.00%), highlighting the substantial uncertainty of this class-specific estimate. The confusion matrices for both evaluations are shown in Figure 2.

The confusion matrices showed a predominantly diagonal distribution in both evaluations (Figure 2). In development, the diagonal proportions were 75.0% for Normal intelligibility, 86.4% for Reduced intelligibility, and 95.2% for Poor intelligibility, with most errors occurring between adjacent categories. In the held-out evaluation, Normal intelligibility and Reduced intelligibility both showed 100% correct classification, whereas 81.3% of Poor intelligibility cases were correctly classified and the remaining 18.8% were predicted as Reduced intelligibility, indicating that residual confusion was concentrated at the boundary between Reduced intelligibility and Poor intelligibility.

The mean outer-fold training and validation accuracies of the main pipeline were 96.00% and 89.94%, respectively. After refitting the selected model on all development patients, training accuracy was 99.00%, compared with 86.96% on the held-out cohort, corresponding to a 12.04-percentage-point gap. The wide confidence intervals for the held-out performance metrics underscore the limited precision of estimates derived from only 23 patients, particularly for the sparsely represented Reduced intelligibility category. Held-out accuracy intervals were calculated using the Wilson method, whereas intervals for the remaining metrics were estimated using 10,000 class-stratified patient-bootstrap resamples. These bootstrap intervals did not account for model-training or selection uncertainty.

As an auxiliary comparison, collapsing predictions from a preceding four-class model into the same three categories yielded 87.00% development accuracy and a macro-F1 of 0.8282. Direct three-class training corrected five previous errors and introduced two new errors. The paired-bootstrap 95% interval for the accuracy difference was −2.0 to +8.0 percentage points, and the macro-F1 difference interval was −0.0483 to +0.1283; both included zero. Because the two pipelines had been optimized for different original objectives, these findings do not establish superiority of direct three-class training.

### 4.3. Comparison of Classification Algorithms and Pretrained Speech Encoders

#### 4.3.1. Classifier Comparison

Among the evaluated configurations, LR yielded the highest observed development accuracy and macro-F1 and also retained the highest held-out accuracy and macro-F1 (Table 6). Linear SVM, RBF SVM, and MLP each correctly classified 19 held-out patients, but their macro-F1 values differed because errors were distributed differently across the three classes. These results support retention of LR within the evaluated pipeline but do not establish general superiority of LR over more complex classifiers.

#### 4.3.2. Pretrained Speech Encoder Comparison

Under single-utterance extraction, HuBERT achieved the highest development macro-F1 (0.8221), whereas data2vec Audio achieved the highest observed held-out accuracy (82.61%) and macro-F1 (0.7333) (Table 7). Chinese wav2vec 2.0 produced the lowest scores in this specific frozen-last-layer setting; these findings do not characterize its performance after task-specific fine-tuning or layer selection. The re-extracted HuBERT baseline obtained 86.00% development accuracy and 73.91% held-out accuracy, compared with 90.00% and 86.96% for the original batched pipeline, indicating that extraction implementation is a material reproducibility consideration. The encoder comparison therefore should not be interpreted as a controlled causal comparison of architecture alone.

#### 4.3.3. Comparison with Deep Baseline

To provide an additional comparison with neural-network-based approaches, a ResNet18 classifier was evaluated using 80-dimensional Log-Mel spectrograms derived from the 27 standardized articulation tasks. The network was initialized without pretrained weights, and its hyperparameters were selected using four-fold cross-validation within an 80-patient subset of the development cohort. The selected model was subsequently trained on all 100 development patients and evaluated on the same 23-patient held-out cohort.

As shown in Table 8, the ResNet18 baseline achieved 82.61% accuracy, a macro-F1 of 0.5463, balanced accuracy of 53.33%, and a QWK of 0.6395. In comparison, the primary HuBERT/PCA/logistic-regression pipeline achieved 86.96% accuracy and a macro-F1 of 0.8227. Although the overall accuracies were relatively close, the ResNet18 baseline showed lower class-balanced performance, particularly for Reduced intelligibility, for which neither of the two held-out patients was correctly classified. These results indicate that the proposed conventional machine-learning pipeline achieved higher observed performance than the evaluated ResNet18 baseline, particularly in terms of macro-F1 and balanced accuracy. However, differences in pretraining and hyperparameter selection should be considered when interpreting this comparison.

### 4.4. Paired Model Comparisons

None of the eight paired accuracy comparisons reached a Holm-adjusted *p*-value below 0.05 (Table 9). This represents an absence of sufficient evidence for a difference rather than evidence of equivalence. The tests address paired accuracy rather than macro-F1, and their power is limited by the small number of discordant patients. In particular, the observed held-out advantage of data2vec over the single-utterance HuBERT baseline does not justify replacing the prespecified primary model.

### 4.5. Patient-Level and Speech-Task Analyses

#### 4.5.1. Patient-Level Prediction Patterns

**Figure 3:**
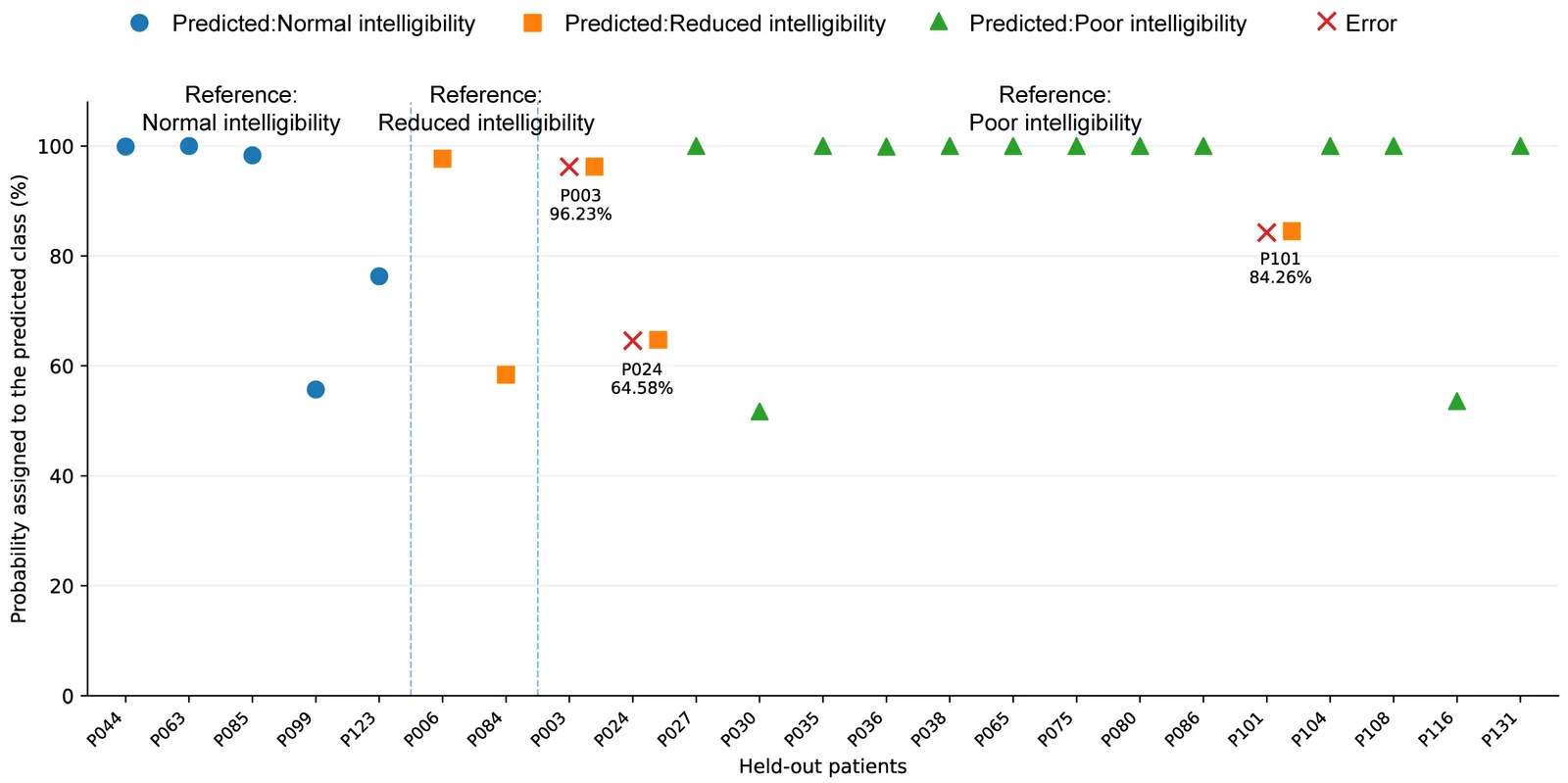
Patient-level probabilities for the locked primary model. Each point represents the probability assigned to the predicted intelligibility class for one held-out patient, and the three misclassified patients are highlighted. Marker shape denotes the predicted class: circles indicate Normal intelligibility, squares indicate Reduced intelligibility, and triangles indicate Poor intelligibility; red × symbols indicate misclassified cases. The vertical dashed lines indicate the boundaries between the three reference intelligibility categories (normal, reduced, and poor intelligibility). The probabilities are raw classifier outputs and are not calibrated clinical confidence estimates.

Figure 3 displays the probability assigned to the predicted intelligibility class for each of the 23 held-out patients. All three misclassified patients had Poor intelligibility reference labels and were predicted as Reduced intelligibility. The predicted Reduced probabilities were 96.23% for P003, 64.58% for P024, and 84.26% for P101. The patient-level error pattern therefore remained confined to the clinical boundary between Reduced and Poor intelligibility rather than extending across the full range from Normal to Poor intelligibility. These examples also illustrate that a high model probability should not be interpreted directly as calibrated clinical certainty.

#### 4.5.2. Speech-Task Replacement Analysis

Task-replacement effects were summarized across all 27 standardized articulation tasks using the predefined class-balanced analysis described in Section 3.11. Equal weighting across the three reference categories prevented the larger Poor intelligibility group from dominating the descriptive task ranking.

**Figure 4:**
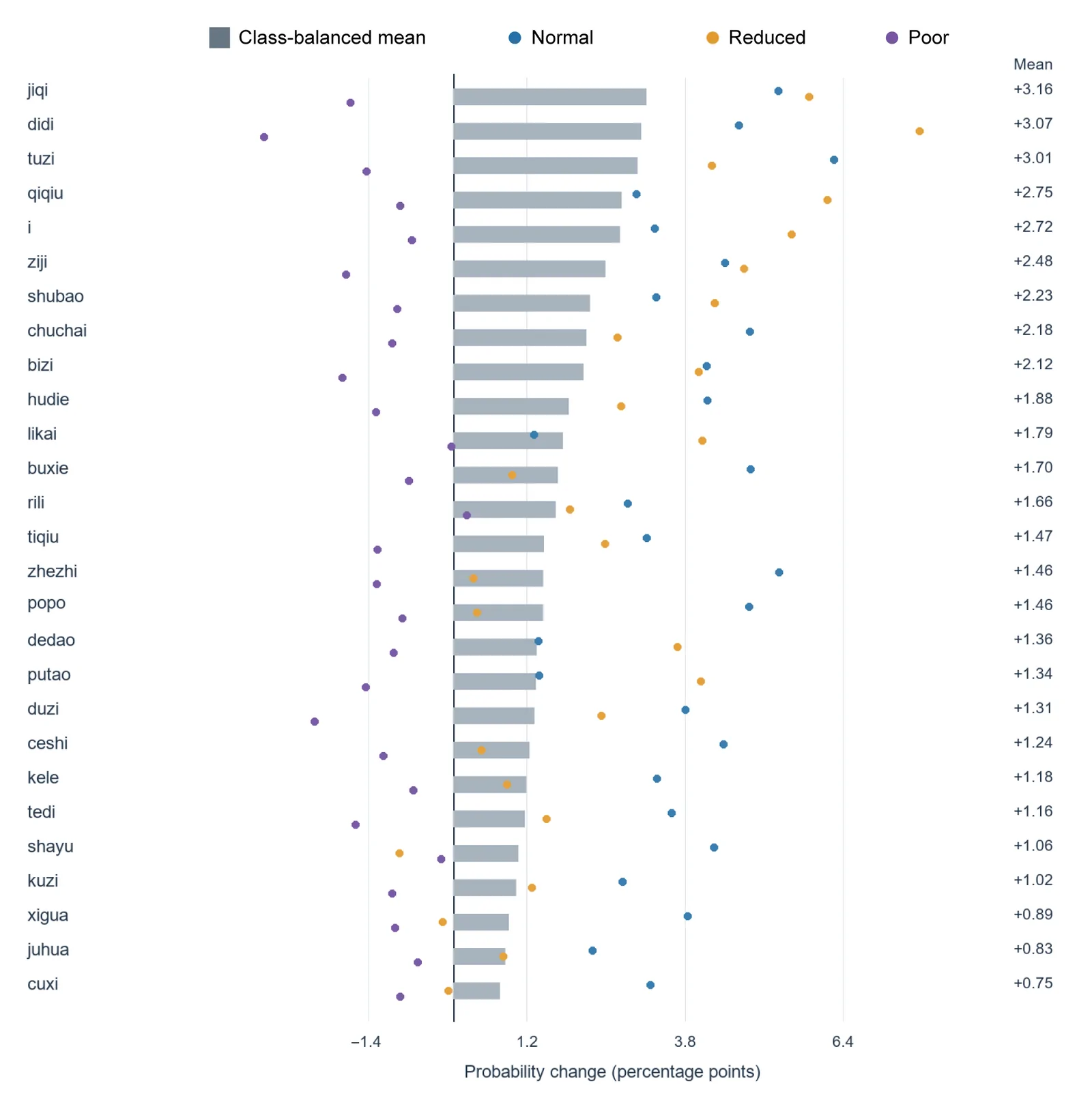
Signed task-replacement effects on the reference-class probability. Gray bars represent the class-balanced mean effect, calculated as the equal-weight mean across the Normal, Reduced, and Poor intelligibility groups, while colored points indicate the corresponding class-specific mean effects. Positive values indicate that replacing a task with its development-cohort mean feature block decreased the probability assigned to the reference class, whereas negative values indicate an increase in the reference-class probability. The 27 articulation tasks are ordered by the class-balanced signed mean, with the corresponding mean values shown on the right. Effects are expressed in percentage points.

The five largest positive class-balanced mean effects were observed for jiqi (3.16 percentage points), didi (3.07), tuzi (3.01), qiqiu (2.75), and i (2.72). Class-specific effects differed across Normal, Reduced, and Poor intelligibility, indicating that task dependence was not uniform across the three clinical categories. These findings identify tasks on which the fitted model relied under the specified replacement baseline; they do not establish that these tasks are causal determinants of intelligibility or clinically superior assessment items. The class-balanced and class-specific task-replacement effects are summarized in Figure 4.

**Figure 5:**
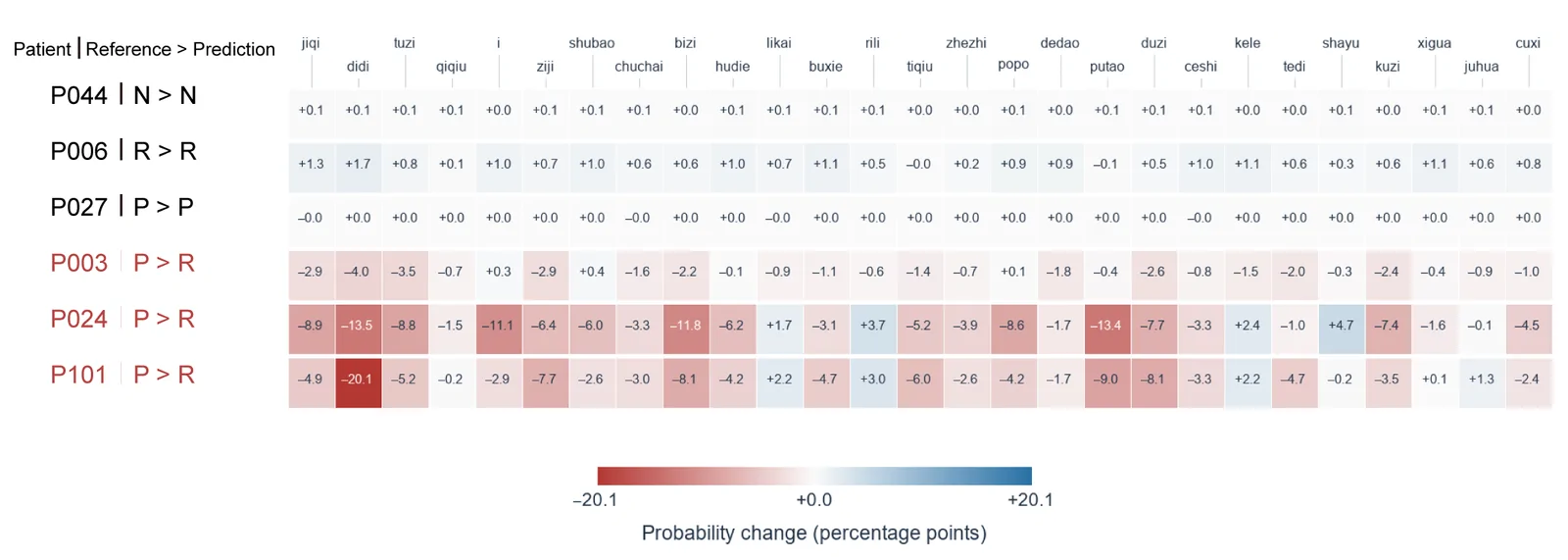
Patient-by-task replacement-effect heatmap for three deterministically selected correctly classified comparison cases and all three errors. Blue indicates a decrease in reference-class probability after replacement, whereas red indicates an increase. A common symmetric scale is used across the matrix. N, R, and P denote Normal, Reduced, and Poor intelligibility, respectively.

For case-level visualization, all three misclassified patients were retained. Correctly classified comparison cases were selected deterministically as the lowest-identifier patient in each reference class: P044 (Normal intelligibility), P006 (Reduced intelligibility), and P027 (Poor intelligibility). For P101, replacing the didi task increased the probability assigned to the Poor intelligibility class by approximately 20.1 percentage points; the corresponding increase was approximately 13.5 percentage points for P024. Both P101 and P024 had Poor intelligibility reference labels but were predicted as Reduced intelligibility, and replacement of didi shifted the model output toward the correct Poor intelligibility category in these two cases. Several correctly classified cases showed near-zero probability changes because their baseline probabilities were close to saturation; therefore, a near-zero change should not be interpreted as evidence that a task contains no predictive information. The complete patient-by-task replacement-effect profiles are shown in Figure 5.

Mean replacement can create feature combinations that are uncommon in real recordings, and task effects depend on the selected reference class and replacement baseline. Correlated tasks may share predictive information, and the effects need not sum to the original prediction. No listening-based phonetic explanation or independent clinician agreement with these task effects was established in this analysis.

### 4.6. Repeated Stratified Nested Cross-Validation

To further assess the robustness of the primary model to patient partitioning, we conducted a supplementary internal validation analysis using five repeats of stratified five-fold outer cross-validation across all 123 patients, comprising 21 patients with Normal intelligibility, 24 with Reduced intelligibility, and 78 with Poor intelligibility. Within each outer training partition, stratified four-fold inner cross-validation was used to select hyperparameters from the original 16-configuration search space, including PCA dimensionality (10 or 20), logistic-regression regularization strength (C ∈ {0.001, 0.01, 0.1, 1}), and class weighting (None or balanced). Hyperparameters were selected based on macro-F1 calculated from pooled inner-validation predictions, followed by the predefined tie-breaking criteria. The original frozen HuBERT feature cache was reused, and all 27 articulation-task recordings from each patient were kept within the same partition. Feature standardization and PCA were fitted exclusively within the corresponding training partitions. Within each repeat, predictions from the five outer folds were aggregated to obtain one out-of-fold prediction for each patient.

Across the five repeats, accuracy was 86.34% ± 1.34 percentage points (range, 85.37–88.62%), macro-F1 was 0.8179 ± 0.0208 (range, 0.7971–0.8527), balanced accuracy was 81.49% ± 2.17 percentage points (range, 79.58–84.97%), and QWK was 0.8686 ± 0.0222 (range, 0.8303–0.8831) (Table 10). Mean class-specific recall across repeats was 81.90% for Normal, 70.00% for Reduced, and 92.56% for Poor intelligibility. Reduced-class recall varied from 58.33% to 79.17%, indicating that the 100% recall observed for the two Reduced patients in the original held-out cohort should not be interpreted as evidence of stable performance for this class.

**Figure 6:**
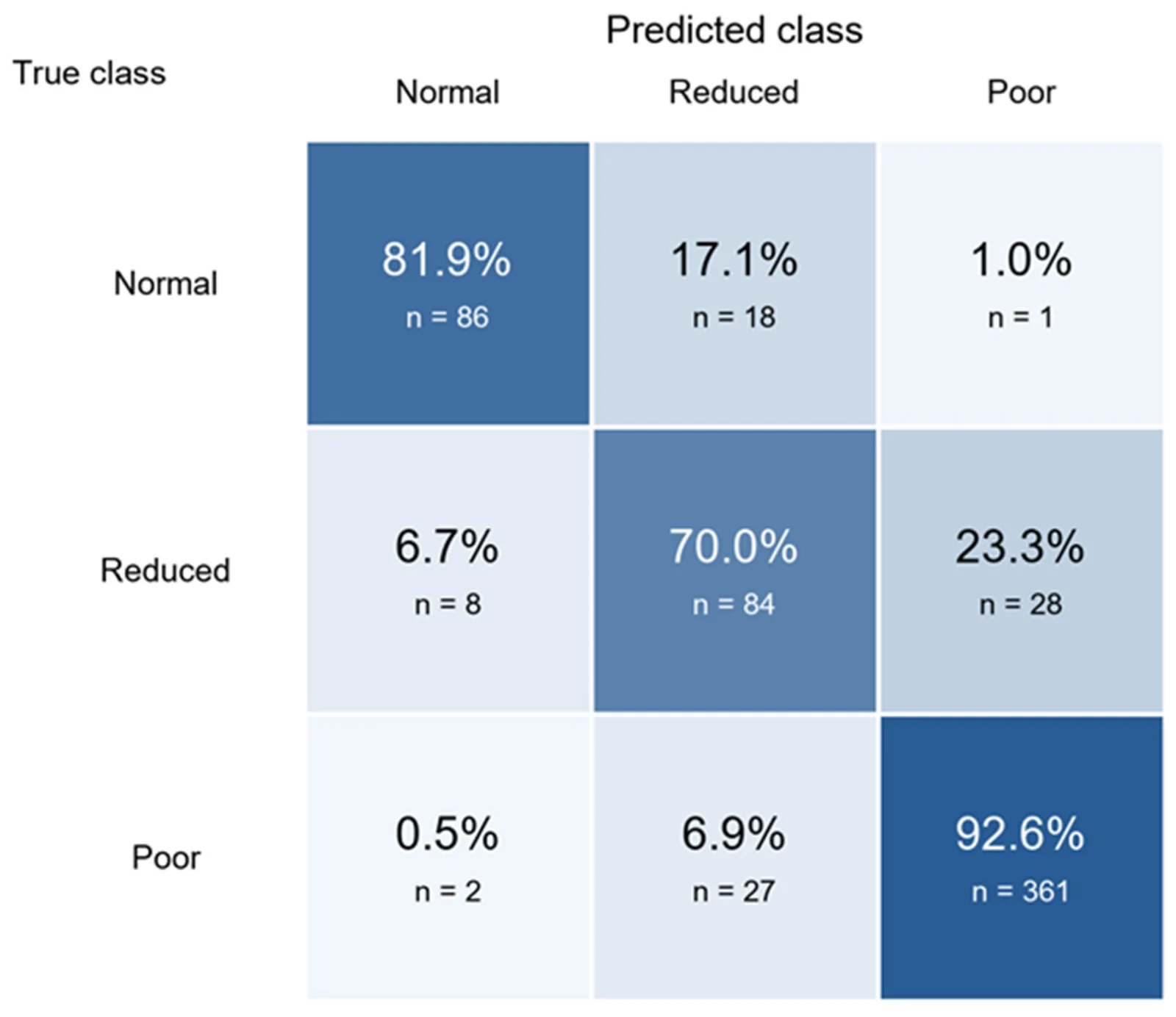
Aggregated out-of-fold confusion matrix across five repeats of stratified nested cross-validation. Percentages are row-normalized. Cell counts represent 615 predictions from 123 unique patients, each evaluated once per repeat. n denotes the number of predictions across repeats. The intensity of the blue shading represents the percentage in each cell, with darker shades indicating higher percentages.

The aggregated confusion matrix is shown in Figure 6. It summarizes 615 out-of-fold predictions from 123 unique patients, with each patient evaluated once in each of the five repeats. The row-normalized results illustrate the differences in class-specific prediction performance, particularly the lower recall for Reduced intelligibility compared with Poor intelligibility.

**Figure 7:**
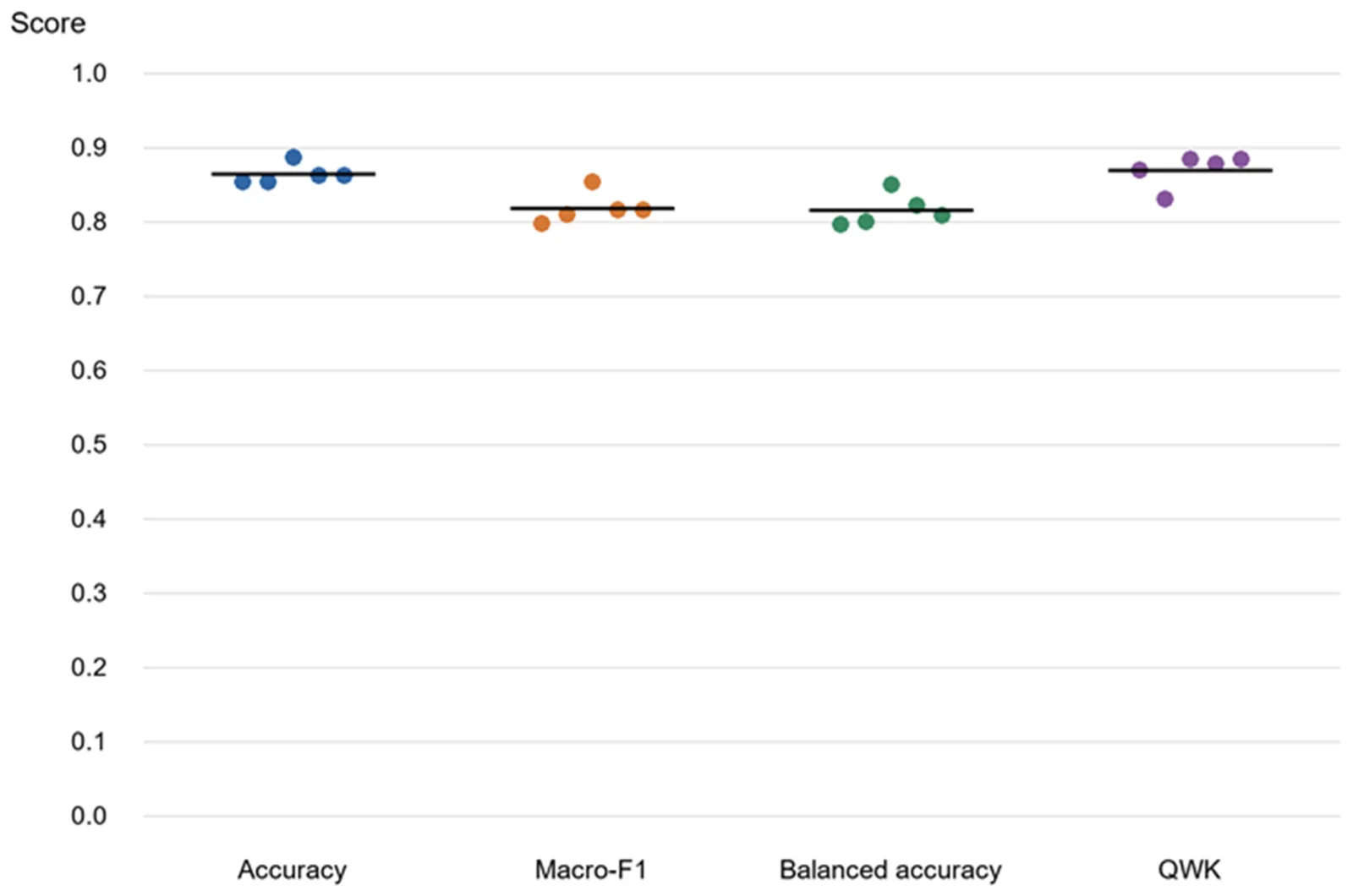
Performance across five repeats of stratified nested cross-validation. Each point represents a metric calculated from the 123 out-of-fold predictions in one complete repeat, and horizontal bars indicate means.

Figure 7 summarizes the performance metrics across the five repeats. Accuracy ranged from 85.37% to 88.62%, while macro-F1 ranged from 0.7971 to 0.8527. These results indicate that overall performance varied within a relatively limited range across the evaluated partitions, although class-specific performance, particularly for Reduced intelligibility, remained less stable.

The original held-out evaluation was retained separately, with an accuracy of 86.96%. However, the 23 patients in the original held-out cohort were also included in this supplementary analysis and contributed to model training in some repeats. Therefore, the repeated cross-validation results represent an internal assessment of sensitivity to patient partitioning rather than a new independent validation or a replacement for the original held-out evaluation. The observed variation across repeats should also be interpreted in light of the limited cohort size, class imbalance, and the reuse of the dataset in previous analyses.

## 5. Discussion

This study evaluated a patient-level framework for classifying overall speech intelligibility in Mandarin-speaking patients with cleft palate using standardized clinical speech recordings. The primary HuBERT/PCA/logistic-regression pipeline achieved 90.00% accuracy with a macro-F1 of 0.8659 in the development cohort and 86.96% accuracy with a macro-F1 of 0.8227 in the held-out cohort. The observed QWK of 0.9043 was consistent with substantial agreement between predicted and reference categories in this small internal cohort. From a clinical grading perspective, all held-out errors were confined to the boundary between Reduced and Poor intelligibility, whereas no direct confusion occurred between Normal and Poor intelligibility. This pattern suggests that the model was more successful in distinguishing clearly different levels of speech performance than in resolving adjacent intelligibility categories. However, the small and imbalanced held-out cohort, particularly the limited number of patients with Reduced intelligibility, requires cautious interpretation of these findings.

Most previous AI studies of cleft palate speech have focused on specific abnormalities such as hypernasality, consonant misarticulation, or velopharyngeal dysfunction [18,19,20,21,22,23,24,25,26,27,28,29,30], whereas relatively few have directly modeled overall speech intelligibility. Earlier work showed that automatic speech recognition-derived measures can reflect intelligibility in children with cleft lip and palate [6], but overall intelligibility remains a multidimensional clinical outcome influenced by the combined effects of resonance, nasal emission, and articulation abnormalities. The present framework therefore uses the patient-level intelligibility category as the prediction target and integrates acoustic information across 27 standardized Mandarin articulation tasks while retaining their predefined correspondence. This organization reflects the structure of the clinical assessment protocol and enables patient-level modeling across multiple phonetic contexts. Task-aligned concatenation and mean pooling differ in both the retention of task-specific feature blocks and their pre-PCA dimensionality (41,472 versus 1536 features). The present comparison therefore does not disentangle the contribution of preserving task identity from that of representation dimensionality, and the observed performance differences should not be attributed solely to task identity.

The comparative experiments also provide several observations regarding model design in a small clinical speech dataset. Among the six evaluated classifier families, regularized logistic regression showed the highest observed development and held-out performance when applied to the archived HuBERT features. This does not imply that logistic regression is generally superior to nonlinear classifiers; rather, it suggests that a relatively low-complexity downstream model can be effective when informative pretrained speech representations are available and sample size is limited. This finding is consistent with the broader trend toward using pretrained or self-supervised encoders as feature extractors in data-scarce pathological-speech applications [14,16,17]. None of the paired classifier comparisons remained significant after Holm correction, and the limited number of discordant held-out cases substantially restricts statistical power. These findings therefore support LR as a suitable low-complexity classifier within the present small-sample clinical setting rather than establishing a general hierarchy among classifier families.

The speech-encoder comparison further highlights the sensitivity of clinical speech modeling to feature-extraction implementation. Under the separate single-utterance extraction protocol, HuBERT achieved the highest development macro-F1, whereas data2vec Audio achieved the highest observed held-out accuracy and macro-F1. Because the evaluated checkpoints differed in pretraining language and corpus composition, these observations should not be interpreted as a controlled comparison of encoder architecture alone. More importantly, the difference between the original batched HuBERT pipeline and the single-utterance HuBERT baseline indicates that feature-extraction implementation can materially affect observed performance in small clinical datasets. This finding emphasizes the importance of fully specified and reproducible preprocessing and representation-extraction procedures when comparing pretrained speech encoders. The observed held-out advantage of data2vec therefore does not justify replacing the prespecified primary model in the present study.

Post hoc task-replacement analysis provided a descriptive view of how individual standardized articulation tasks influenced the fitted model. The heterogeneous task-replacement profiles across patients may reflect differences in how cleft-palate-related speech impairment is expressed across phonetic contexts. The largest class-balanced effects were observed for jiqi, didi, tuzi, qiqiu, and i, while individual patients showed substantially different response patterns. However, these effects should not be interpreted as evidence that specific tasks are clinically superior or causally more important. Mean replacement may generate uncommon feature combinations, correlated tasks may contain overlapping predictive information, and no independent listening-based phonetic interpretation or clinician agreement was available. These findings are therefore better regarded as hypothesis-generating evidence for future phonetic and clinical analyses rather than as a basis for shortening the standardized assessment protocol.

Several limitations should be considered. First, the study was based on a single-center cohort of 123 patients, and the held-out cohort contained only 23 patients with marked class imbalance, including only two patients with Reduced intelligibility. Consequently, the 100% recall observed for Reduced intelligibility is based on only two held-out cases, and class-specific performance estimates remain imprecise. Second, although the reference labels were established through independent assessments and consensus among three experienced clinicians, perceptual intelligibility grading remains inherently subjective, and the substantial but imperfect inter-rater agreement (κ = 0.719) indicates some variability in clinical judgments. Third, the development dataset had been used during earlier experimentation, and the internal held-out cohort does not provide the same level of evidence as an independently collected external or prospective validation cohort. Nested cross-validation controls data leakage within the reported modeling procedure but cannot fully remove bias arising from broader iterative dataset use. Fourth, classifier probabilities were not calibrated as clinical confidence estimates, and the post hoc task-replacement analysis was descriptive rather than causal. Future work should therefore include larger multicenter cohorts, prospective external validation, explicit assessment of annotation reliability, standardized feature-extraction pipelines, probability calibration, and independent clinical interpretation of task-level effects. Under these conditions, automated intelligibility assessment may become a useful adjunct for standardized postoperative follow-up rather than a replacement for specialist perceptual evaluation.

## 6. Conclusions

This study demonstrates the feasibility of automated patient-level classification of overall speech intelligibility in Mandarin-speaking patients with cleft palate using standardized articulation recordings and pretrained speech representations. Using a structured patient-level representation across 27 standardized articulation tasks, the HuBERT/PCA/logistic-regression pipeline achieved 90.00% accuracy in development and 86.96% accuracy in the held-out cohort. All held-out misclassifications occurred between Reduced and Poor intelligibility, with no direct confusion between Normal and Poor intelligibility. The comparison with mean pooling supports an advantage in observed performance for the overall task-aligned representation, but does not establish an independent benefit of task identity. Importantly, only two held-out patients had Reduced intelligibility, so class-specific performance estimates for this subgroup remain uncertain. These findings support the potential of automated speech analysis as an objective adjunct to postoperative cleft palate speech assessment.

## Figures and Tables

**Table 1:** Distribution of speech intelligibility categories in the HCPSI dataset.

Subset	Normal Intelligibility	Reduced Intelligibility	Poor Intelligibility	Total
Development	16	22	62	100
Held-out test	5	2	16	23

**Table 2:** Demographic characteristics of the HCPSI cohort.

Characteristic	Development (*n* = 100)	Held-Out (*n* = 23)	Total (*n* = 123)	*p*
Age, median (range), years	14 (4–37)	12 (4–35)	14 (4–37)	0.582
Male, *n* (%)	48 (48.0)	15 (65.2)	63 (51.2)	0.136
Female, *n* (%)	52 (52.0)	8 (34.8)	60 (48.8)	

Note: *p* values compare the development and held-out cohorts. Age was compared using the Mann–Whitney U test, and sex distribution was compared using the chi-square test.

**Table 3:** Patient allocation across the five outer folds and corresponding inner cross-validation splits.

Outer Fold	Outer Training (*n*)	Outer Validation (*n*)	Inner Training (*n*)	Inner Validation (*n*)
F1	80	20	61, 60, 60, 59	19, 20, 20, 21
F2	81	19	61, 61, 61, 60	20, 20, 20, 21
F3	80	20	60, 61, 60, 59	20, 19, 20, 21
F4	80	20	60, 61, 60, 59	20, 19, 20, 21
F5	79	21	59, 60, 59, 59	20, 19, 20, 20

Note: Inner training and validation counts correspond to the four inner splits within each outer iteration, in the order of the remaining fixed folds. All splits were patient-disjoint, and the 23 held-out test patients were excluded from the nested cross-validation procedure.

**Table 4:** Comparison of task-aligned and task-agnostic mean-pooling representations.

Evaluation	Representation	Accuracy (%)	Macro-F1	Balanced Accuracy (%)	QWK
Development	Task-aligned	90.00	0.8659	85.51	0.8519
Development	Mean pooling	82.00	0.7513	74.21	0.7778
Held-out	Task-aligned	86.96	0.8227	93.75	0.9043
Held-out	Mean pooling	73.91	0.7012	82.92	0.7953

Note: Development performance was obtained from nested cross-validation out-of-fold predictions. Both approaches used the same patient partitions and downstream model-selection procedure.

**Table 5:** Performance of the primary HuBERT/PCA/logistic-regression pipeline.

Subset	Accuracy (%)	Macro-F1	Balanced Accuracy (%)	QWK
Development (nested OOF)	90.00	0.8659	85.51	0.8519
Held-out test	86.96 [67.87, 95.46]	0.8227 [0.7231, 1.0000]	93.75 [87.50, 100.00]	0.9043 [0.8078, 1.0000]

Note: Bracketed values indicate 95% confidence intervals for the held-out cohort.

**Table 6:** Classifier comparison using archived HuBERT features. Development scores are nested out-of-fold estimates; held-out scores use full-development refits.

Classifier	Development Accuracy (%)	Development Macro-F1	Held-Out Accuracy (%)	Held-Out Macro-F1
Logistic regression	90.00	0.8659	86.96	0.8227
Linear SVM	87.00	0.8316	82.61	0.7085
RBF SVM	83.00	0.7749	82.61	0.7333
Random forest	80.00	0.7420	69.57	0.6739
KNN	79.00	0.7033	78.26	0.7302
MLP	86.00	0.8160	82.61	0.7085

**Table 7:** Frozen feature-extractor comparison using single-utterance extraction and PCA/LR. These results use a separate extraction protocol from the primary archived pipeline.

Encoder	Development Accuracy (%)	Development Macro-F1	Held-Out Accuracy (%)	Held-Out Macro-F1
Chinese HuBERT	86.00	0.8221	73.91	0.7012
Chinese wav2vec 2.0	69.00	0.5621	56.52	0.4691
WavLM Base+	84.00	0.7619	73.91	0.6222
data2vec Audio Base	80.00	0.7329	82.61	0.7333

**Table 8:** Held-out performance comparison between the primary pipeline and the ResNet18 deep-learning baseline.

Model	Accuracy (%)	Macro-F1	Balanced Accuracy (%)	QWK
HuBERT + PCA + LR	86.96	0.8227	93.75	0.9043
Log-Mel ResNet18	82.61	0.5463	53.33	0.6395

**Table 9:** Exploratory exact McNemar tests on paired held-out correctness. R-only and A-only denote patients correctly classified only by the reference or alternative model, respectively; Holm correction covers all eight comparisons.

Round	Reference	Alternative	R-Only	A-Only	Exact *p*	Holm-Adjusted *p*
1	LR	Linear SVM	2	1	1.0000	1.0000
1	LR	RBF SVM	2	1	1.0000	1.0000
1	LR	Random forest	4	0	0.1250	1.0000
1	LR	KNN	2	0	0.5000	1.0000
1	LR	MLP	2	1	1.0000	1.0000
2	HuBERT	wav2vec 2.0	6	2	0.2891	1.0000
2	HuBERT	WavLM	2	2	1.0000	1.0000
2	HuBERT	data2vec	1	3	0.6250	1.0000

**Table 10:** Performance across five repeats of stratified nested cross-validation.

Metric	Mean ± SD	Range Across Repeats
Accuracy (%)	86.34 ± 1.34	85.37–88.62
Macro-F1	0.8179 ± 0.0208	0.7971–0.8527
Balanced accuracy (%)	81.49 ± 2.17	79.58–84.97
QWK	0.8686 ± 0.0222	0.8303–0.8831
Recall: Normal (%)	81.90 ± 8.52	71.43–90.48
Recall: Reduced (%)	70.00 ± 8.01	58.33–79.17
Recall: Poor (%)	92.56 ± 1.07	91.03–93.59

Note: Each repeat represents one complete stratified five-fold outer cross-validation run. Standard deviations describe variation across the five repeats and are not 95% confidence intervals. Values are reported as mean ± standard deviation (SD) and range across the five repeats. Percentage-based metrics are expressed in %, with SDs expressed in percentage points.

## Data Availability

The data supporting the findings of this study are available from the corresponding author upon reasonable request.

## Abbreviations

The following abbreviations are used in this manuscript:

AI	Artificial intelligence
ASR	Automatic speech recognition
CNN	Convolutional neural network
CP	Cleft palate
HCPSI	Huaxi Cleft Palate Speech Intelligibility
KNN	k-nearest neighbors
LR	Logistic regression
LSTM	Long short-term memory
MFCC	Mel-frequency cepstral coefficient
MLP	Multilayer perceptron
PCA	Principal component analysis
QWK	Quadratic-weighted kappa
RBF	Radial basis function
SVM	Support vector machine
